# Clinical predictors of the presence of obstructive sleep apnea in patients with hypertrophic cardiomyopathy

**DOI:** 10.1038/s41598-021-93039-5

**Published:** 2021-06-29

**Authors:** Haobo Xu, Juan Wang, Jiansong Yuan, Chao Guo, Fenghuan Hu, Weixian Yang, Lei Song, Xiaoliang Luo, Rong Liu, Jingang Cui, Shengwen Liu, Yushi Chun, Yunhu Song, Shubin Qiao

**Affiliations:** Department of Cardiology, Fuwai Hospital, National Center for Cardiovascular Diseases, Chinese Academy of Medical Sciences, Peking Union Medical College, A 167 Beilishi Road, Xicheng District, Beijing, 100037 People’s Republic of China

**Keywords:** Risk factors, Cardiac hypertrophy, Epidemiology, Respiratory tract diseases

## Abstract

Obstructive sleep apnea (OSA) is much common and associated with worse clinical outcomes in patients with hypertrophic cardiomyopathy (HCM), however, the diagnosis of OSA in HCM is still insufficient. We aim to investigate the clinical predictors of OSA in a large series of patients with HCM. A total of 589 patients with HCM who underwent sleep evaluations were retrospectively enrolled. Data from clinical characteristics and polysomnography studies were recorded. OSA was present in 346 patients (58.7%). Patients who had OSA were older, more likely to be male and had more clinical comorbidities such as hypertension, atrial fibrillation and cardiac remodeling. Multivariate logistic analyses showed that male, age, body mass index, hypertension and left ventricular outflow tract obstruction were significant factors associated with OSA. The area under the ROC curve (AUC) was 0.78 (95% CI 0.74–0.82; P < 0.001). These factors were also able to identify moderate to severe OSA with an AUC of 0.77 (95% CI 0.73–0.81; P < 0.001). These findings suggest that identifying HCM patients with high risk for OSA is feasible using characteristics from clinical practices and clinicians should have no hesitate to conduct sleep test in these patients.

## Introduction

Hypertrophic cardiomyopathy (HCM) is one of the most common genetic cardiovascular diseases^1^. Despite improvements of pharmacological, surgical and interventional therapies in management of HCM, patients are at risk for a number of adverse events such as progressive heart failure, arrhythmias and sudden death^2–4^. Recently, increasing evidences from our works^5,6^ and others^7,8^ showed that obstructive sleep apnea (OSA) is extremely common among patients with HCM, with a prevalence ranging from 32 to 71%. OSA is independently associated with cardiac remodeling^9^, atrial fibrillation^5^ and ventricular arrhythmias^10^ which are risk factors for cardiac death in patients with HCM. Therefore, screening and management of OSA have great potential to improve clinical outcomes in HCM.

However, there is still a lack of recognition of OSA in daily clinical practices and we did not find any mention of OSA in the text of HCM guidelines^1,11^. Identifying clinical predictors of OSA in patients with HCM could help increase the diagnosis as well as the awareness of OSA in HCM. In that case, we performed the present study to investigate the clinical predictors of OSA in a large series of patients with HCM. A total of 589 patients with HCM who underwent an overnight diagnostic sleep study at Fuwai Hospital were included. We hope our results could help identify patients at high risk for OSA among HCM.

## Results

### Population characteristics

Of the included patients, 346 (58.7%) were diagnosed with OSA and the median AHI value of the whole study population was markedly elevated (7.4 events/h, interquartile range [IQR] 1.9–18.9 events/h). Table 1 shows the baseline characteristics of the whole study population as well as patients grouped according to the presence of OSA. Patients who had OSA were older, more likely to be male, had a higher BMI, be smokers, and had more comorbidities such as hypertension, diabetes, coronary heart disease as well as stroke. The prevalence of atrial fibrillation was significant higher in patients with OSA than those without. Levels of fasting glucose and creatinine were increased in patients with OSA. The level of N-terminal pro-B-type natriuretic peptide was elevated in the whole study populations and was relatively low in patients with OSA compared with patients without OSA. Echocardiographic studies showed that patients with OSA had lower prevalence of LVOT obstruction. The interventricular septum thickness was also lower in patients with OSA. Whereas, patients with OSA were associated with enlarged left atrial diameter and left ventricular end-diastolic diameter. The percent of patients taking calcium channel blockers and angiotensin-converting enzyme inhibitors or angiotensin II receptor blockers were higher in patients with OSA.

**Table 1:** Clinical characteristics of patients with HCM grouped according to the presence of OSA.

Variables	All patients (n = 589)	No OSA (n = 243)	OSA (n = 346)	*P*-value
Male	423 (71.8)	157 (64.6)	266 (76.9)	0.001
Age (y)	50.5 ± 12.8	45.7 ± 13.7	53.8 ± 11.1	< 0.001
BMI (kg/m^2^)	26.2 ± 3.7	24.7 ± 3.3	27.2 ± 3.6	< 0.001
Cigarette use	278 (47.2)	92 (37.9)	186 (53.8)	< 0.001
Hypertension	280 (47.5)	72 (29.6)	208 (60.1)	< 0.001
Systolic blood pressure (mmHg)	129.9 ± 19.5	124.5 ± 16.5	133.7 ± 20.5	< 0.001
Diastolic blood pressure (mmHg)	77.9 ± 12.9	74.1 ± 11.4	80.5 ± 13.2	0.110
Diabetes	79 (13.4)	21 (8.6)	58 (16.8)	0.004
Coronary heart disease	84 (14.3)	22 (9.1)	62 (17.9)	0.002
Stroke	41 (7.0)	9 (3.7)	32 (9.2)	0.009
Atrial fibrillation	118 (20.0)	31 (12.8)	87 (25.1)	< 0.001
Ventricular tachycardia	81 (13.8)	29 (11.9)	52 (15.0)	0.283
NYHA class II–III	397 (67.4)	154 (63.4)	243 (70.2)	0.081
Syncope	70 (11.9)	27 (11.1)	43 (12.4)	0.627
Family history of HCM	61 (10.4)	32 (13.2)	29 (8.4)	0.060
Heart rate (rpm)	71.0 ± 11.1	70.8 ± 10.5	71.2 ± 11.5	0.095
**Biochemical tests**				
Fasting glucose (mmol/L)	5.1 ± 1.4	4.8 ± 1.2	5.3 ± 1.5	< 0.001
Total cholesterol (mmol/L)	4.4 ± 1.0	4.4 ± 0.9	4.4 ± 1.0	0.572
LDL-C (mmol/L)	2.7 ± 0.8	2.7 ± 0.8	2.7 ± 0.8	0.577
HDL-C (mmol/L)	1.1 ± 0.4	1.1 ± 0.4	1.1 ± 0.4	0.055
Hs-CRP (mg/L)	1.1 (0.5–2.4)	0.8 (0.4–1.7)	1.5 (0.7–2.9)	< 0.001
Creatine (µmol/L)	85.3 ± 18.0	82.4 ± 15.8	87.3 ± 19.1	0.001
Uric acid (µmol/L)	394.6 ± 101.8	385.0 ± 101.2	401.4 ± 101.7	0.054
NT-proBNP (pmol/L)	787.4 (297.0–1765.0)	987.0 (465.6–2017.0)	629.4 (225.6–1565.5)	< 0.001
**Echocardiographic data**				
LVOT obstruction	348 (59.1)	166 (68.3)	182 (52.6)	< 0.001
LVOT gradient	30.0 (0.0–66.0)	44.0 (0.0–75.0)	20.5 (0.0–61.3)	< 0.001
SAM	310 (52.6)	145 (59.7)	165 (47.7)	0.004
LAD (mm)	42.8 ± 7.2	42.1 ± 6.5	43.4 ± 7.6	0.031
LVEDD (mm)	45.3 ± 6.2	43.8 ± 6.3	46.5 ± 5.9	< 0.001
IVST (mm)	17.7 ± 4.8	18.6 ± 5.0	17.2 ± 4.6	0.001
LVEF (%)	65.9 ± 8.8	66.6 ± 8.6	65.4 ± 8.9	0.110
**Medical therapy**				
β-Blockers	512 (86.9)	219 (90.1)	293 (84.7)	0.054
CCBs	156 (26.5)	41 (16.9)	115 (33.2)	< 0.001
ACEIs/ARBs	176 (29.9)	50 (20.6)	126 (36.4)	< 0.001
Spirolactone	68 (11.5)	29 (11.9)	39 (11.3)	0.804
Class III antiarrhythmic drugs	77 (13.1)	33 (13.6)	44 (12.7)	0.760
Anticoagulation therapy	88 (14.9)	30 (12.3)	58 (16.8)	0.139

Values are presented as mean ± standard deviation, as median (interquartile range), or as n (%).

HCM: hypertrophic cardiomyopathy; OSA: obstructive sleep apnea; BMI: body mass index; NYHA: New York Heart Association; LDL-C: low-density lipoprotein cholesterol; HDL-C: high-density lipoprotein cholesterol; hs-CRP, high-sensitivity C-reactive protein; NT-proBNP: N-terminal pro-B-type natriuretic peptide;LVOT: left ventricular outflow tract; SAM, systolic anterior motion; LAD: left atrial diameter; LVEDD: left ventricular end-diastolic dimension; IVST: Interventricular septum thickness; LVEF: left ventricular ejection fraction; PSG: polysomnography; AHI: apnea hypopnea index; ODI: oxygen desaturation index; SaO_2_: oxygen saturation; TST: total sleep time; HR: heart rate; CCBs: calcium channel blockers; ACEIs: angiotensin-converting enzyme inhibitors; ARBs: angiotensin II receptor blockers.

### Sleep parameters

Data of sleep examinations are summarized in Table 2. The value of AHI, oxygen desaturation index, longest apnea/hypopnea time, total sleep time spent with oxygen saturation (SaO_2_) < 90%, snoring time ratio and heart rate during sleep were all significantly higher in patients with OSA than those without OSA. Lowest SaO_2_ and mean SaO_2_ reflecting oxygen level were decreased in patients with OSA.

**Table 2:** Sleep data of patients with HCM grouped by the presence of OSA.

Variables	All patients (n = 589)	No OSA (n = 243)	OSA (n = 346)	*P*-value
AHI (events/h)	7.4 (1.9–18.9)	1.5 (0.6–2.8)	16.7 (9.0–31.0)	< 0.001
ODI (events/h)	7.4 (2.6–18.8)	2.1 (1.0–4.1)	15.9 (9.0–30.0)	< 0.001
Longest apnea/hypopnea time (s)	57.0 (37.0–79.1)	35.6 (24.1–51.3)	69.7 (53.8–91.7)	< 0.001
Lowest SaO_2_ (%)	83.4 ± 7.6	88.0 ± 4.0	80.2 ± 7.9	< 0.001
Mean SaO_2_ (%)	93.7 ± 2.1	94.5 ± 1.7	93.2 ± 2.1	< 0.001
TST with SaO_2_ < 90% (%)	1.6 (0.0–8.3)	0.0 (0.0–1.4)	5.3 (1.1–13.5)	< 0.001
Snoring time ratio (%)	5.2 (1.0–12.5)	3.0 (0.5–6.5)	8.2 (2.3–15.4)	< 0.001
Mean HR during sleep (rpm)	60.7 ± 7.5	59.9 ± 7.3	61.2 ± 7.6	0.040
Supine sleep time (min)	208.7 ± 101.0	220.4 ± 110.0	200.4 ± 93.7	0.018
TST (min)	468.8 ± 87.2	486.7 ± 94.2	456.3 ± 79.7	< 0.001

Values are presented as mean ± standard deviation or as median (interquartile range).

HCM: hypertrophic cardiomyopathy; OSA: obstructive sleep apnea; AHI: apnea hypopnea index; ODI: oxygen desaturation index; SaO_2_: oxygen saturation; TST: total sleep time; HR: heart rate.

### Association between clinical characteristics and presence of OSA

**Figure 1:**
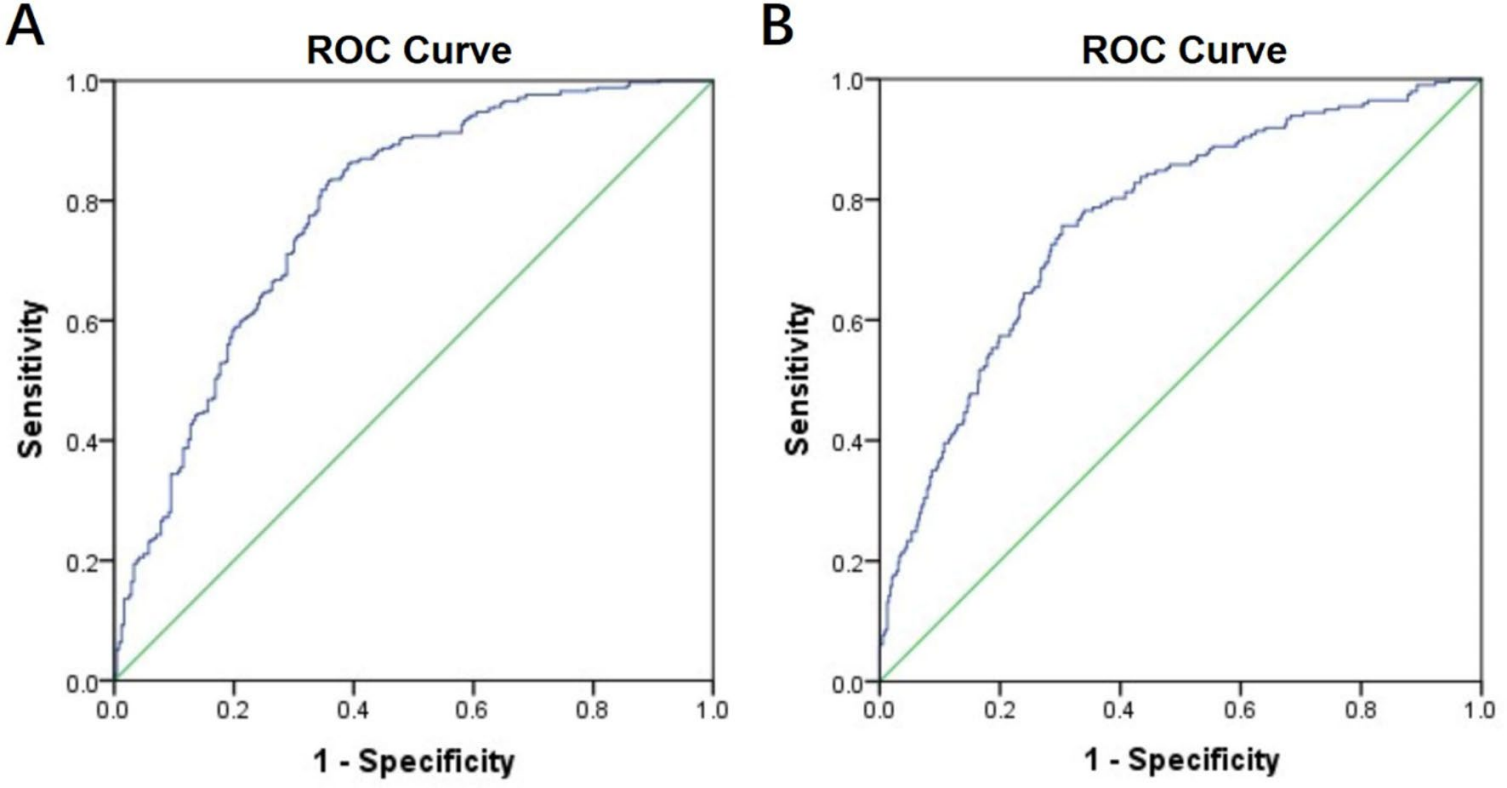
Receiver-operator characteristic (ROC) curve to identify obstructive sleep apnea (OSA). ROC curves for the clinical factors including male, age, body mass index, hypertension and left ventricular outflow tract obstruction to identify the presence of OSA (**A**) as well as the presence of moderate to severe OSA (**B**).

In univariate analyses, male sex, age, BMI, cigarette use, hypertension, diabetes, coronary heart disease, stroke, atrial fibrillation, creatine, LVOT obstruction, LAD, LV end-diastolic diameter, IVST were found to be significantly associated with the presence of OSA (Table 3). After enrolling these covariates in multivariate analyses, male (OR, 1.67; 95% CI 1.08–2.57), age (OR, 1.06; 95% CI 1.04–1.08), BMI (OR, 1.24; 95% CI 1.16–1.31), hypertension (OR, 1.78; 95% CI 1.19–2.66) and LVOT obstruction (OR, 0.59; 95% CI 0.40–0.87) were significant factors associated with OSA. The ROC curve for assessing the ability of these factors to identify OSA is shown in Fig. 1A. The area under the ROC curve (AUC) was 0.78 (95% CI 0.74–0.82; *P* < 0.001). We then tested the ability of these factors to identify the presence of moderate to severe OSA (Fig. 1B) and the AUC was 0.77 (95% CI 0.73–0.81; *P* < 0.001).

**Table 3:** Logistic regression between OSA and significant clinical variables from univariate analysis.

Variables	Univariate		Multivariate	
	OR (95%CI)	*P*-value	OR (95%CI)	*P*-value
Male	1.82 (1.27–2.62)	0.001	1.67 (1.08–2.57)	0.021
Age (y)	1.05 (1.04–1.07)	< 0.001	1.06 (1.04–1.08)	< 0.001
BMI (kg/m^2^)	1.24 (1.17–1.31)	< 0.001	1.24 (1.16–1.31)	< 0.001
Cigarette use	1.91 (1.37–2.67)	< 0.001		
Hypertension	3.58 (2.52–5.08)	< 0.001	1.78 (1.19–2.66)	0.005
Diabetes	2.13 (1.25–3.61)	0.005		
Coronary heart disease	2.19 (1.31–3.68)	0.003		
Stroke	2.65 (1.24–5.66)	0.012		
Atrial fibrillation	2.30 (1.47–3.60)	< 0.001		
Creatine (µmol/L)	1.02 (1.01–1.03)	0.001		
LVOT obstruction	0.52 (0.37–0.73)	< 0.001	0.59 (0.40–0.87)	0.009
LAD (mm)	1.03 (1.00–1.05)	0.032		
LVEDD (mm)	1.08 (1.05–1.11)	< 0.001		
IVST (mm)	0.94 (0.91–0.98)	0.001		
CCBs	2.45 (1.64–3.67)	< 0.001		
ACEIs/ARBs	2.21 (1.51–3.23)	< 0.001		

Data are presented as odds ratio (95% confidence interval).

OR: odds ratio; CI: confidence interval; OSA: obstructive sleep apnea; BMI: body mass index; LVOT: left ventricular outflow tract; LAD: left atrial diameter; LVEDD: left ventricular end-diastolic dimension; IVST: interventricular septum thickness; CCBs: calcium channel blockers; ACEIs: angiotensin-converting enzyme inhibitors; ARBs: angiotensin II receptor blockers.

## Discussion

Our study was designed to determine the clinical characteristics that may help to identify OSA among HCM patients. Our results showed that OSA (58.7%) was much common in patients with HCM. Patients with OSA had a higher grade of cardiac remodeling such as enlarged LAD and left ventricular end-diastolic diameter and higher prevalence of atrial fibrillation. In multivariate analysis, male, age, BMI, hypertension and LVOT obstruction were significant factors associated with OSA. In addition, these factors also had a good ability to identify moderate to severe OSA.

It has been reported that OSA is extremely common in HCM. In our study, the prevalence of OSA was 58.7% which is in agreement with previous studies showing a prevalence ranging from 32 to 71%. A number of studies have implicated OSA as an independent risk factor for cardiovascular diseases in the general population. In patients with HCM, increasing evidences also indicated that OSA is independently associated with cardiac remodeling and arrythmias. For example, our previous studies showed that severity of OSA is independently associated with the prevalence of atrial fibrillation. In the present study, patients with OSA were older, had a higher BMI and had more clinical comorbidities such as hypertension, diabetes and coronary heart disease which are all risk factors for adverse cardiac outcomes in HCM. Therefore, these results suggested that screening and treatment of OSA in HCM is of potential ability to improve prognosis. However, there is a lack of recognition of OSA among HCM in clinical practices. There are several potential explanations. First, cardiologists are paying more attentions on the reduction of LVOT obstruction and prevention of sudden cardiac death among HCM but not evaluation of sleep quality. Second, the high prevalence of OSA in this population is only recently been demonstrated and the impacts of OSA on long-term prognosis is still unknown. More evidence is still needed to demonstrate the benefits of OSA treatment in HCM. Third, patients with HCM and OSA do not exhibit the typical features of OSA patients in general populations such as obesity, tiredness and excessive daytime sleepiness^12^. For instance, in our study, the mean BMI levels of patients with OSA was 27.2 kg/m^2^ which was much lower than typical OSA patients. This might also abort the attempts of sleep test from clinicians. Based on above reasons, clinical predictors that can be easily used to detect the patients with high risk for OSA is of great need.

Identification of clinical predictors of OSA among HCM had been explored before by Nerbass et al.^12^. In their study, they found that Berlin questionnaire, which has been previously validated to recognize patients with OSA in general populations, was not useful in recognizing OSA among HCM. These results suggested that classical risk factors for OSA in general population might not useful for detecting OSA in patients with HCM. Nerbass et al. also showed that only age ≥ 45 years and the presence of atrial fibrillation were predictors of OSA after adjusting related confounders. However, the study population in their study was 90 patients which was relatively low and might not guarantee the accuracy and precision of regression model and could result in overfitting. In our study, the number of HCM cohort was the largest to date about HCM and OSA and therefore, more covariates could be enrolled in regression model. We found that male sex, age, BMI, hypertension and LVOT obstruction were significant factors associated with OSA after controlling for related covariates. These factors are all from clinical characters and are easy to acquire in clinical practices. The AUC is 0.78 (95% CI 0.74–0.82) and it can be considered in this setting as a good classifier considering that all factors or inputs are clinical acquired and do not involve any polysomnographic variable.

The coefficients of male sex, age, BMI and hypertension are all positive, meaning that they all are positively associated with OSA. For instance, the odds of being a OSA patient is increased by 67% in male compared with female and by 78% in patients with hypertension compared with those without. Our results were in line with previous studies showing that the prevalence of OSA increases with age and is approximately twice as common in men as in women in general populations^13,14^. Taken together, our results suggested that risk factors for OSA that found in general populations were also critical in patients with HCM despite the facts that patients with OSA in HCM do not exhibit the typical features in general populations. Atrial fibrillation, which was found to be a predictor of OSA by Nerbass et al., was not significant in our study. We have previously showed that OSA is independently associated with atrial fibrillation in patient with HCM^5^. Therefore, atrial fibrillation is more likely to be a consequence of OSA but not a causation. In this case, atrial fibrillation was only significant in univariate analysis but not after controlling for other covariates. Another risk factor we found was LVOT obstruction and it was negatively associated with OSA which meant that patients with non-obstructive HCM were more likely to have OSA compared with patients with obstructive HCM. This finding was interesting because a hemodynamic trait in HCM could act as a predictor of OSA. One possible explanation is that, unlike patients with obstructive HCM who are usually diagnosed at young age, patients with non-obstructive HCM are usually asymptomatic^15^ and more likely to be diagnosed at older age which increases the risk of OSA. Another reason is that patients with non-obstructive HCM usually develop cardiac dysfunction manifesting as preserved ejection fraction and diastolic dysfunction which aggravates water-sodium retention and subsequently overnight rostral fluid shift to the neck contributing to upper airway obstruction^8,16,17^. Nevertheless, considering the high prevalence of OSA in HCM, suspension and screening of OSA should not be differed between different HCM phenotypes. Severity of OSA is important for the risk stratification and determination of treatment strategies in patients with OSA^18^. For example, patients with moderate to severe OSA had significantly increased cardiovascular risk compared with patients with mild OSA and are indicated to receive continuous positive airway pressure therapy^19^. In our study, the clinical predictors we found were not only able to identify OSA but also to identify moderate to severe OSA. These results additionally suggested that the predictors we found were useful in clinical practices and may help cardiologists actively screening for OSA among patients with HCM.

Our study has several limitations. First, the generalizability of our findings was limited by the single center as well as the selection bias in study populations, as they presented to a tertiary medical center for their care and were more symptomatic. Second, all the patients in our study received only one night of sleep examination. Previous studies showed that there is a considerable night-to-night variability and single-night sleep study is prone to misdiagnosis and miscategorize the severity of OSA^20,21^. In that case, repeated measurements will help to improve the accuracy of diagnosis and patient categorization. Third, the clinical predictors we found was only able to identify the prevalence of OSA but not to predict the incidence of OSA. Therefore, more work is needed to investigate the usefulness of these factors to predict the incidence of OSA.

In conclusion, our study showed that OSA is highly prevalent in patients with HCM. Clinical factors such as male, age, BMI, hypertension and LVOT obstruction are useful to identify the patients at high risk for OSA. Future studies to include larger populations of patients with HCM should be undertaken to confirm these findings.

## Materials and methods

### Study population

**Figure 2:**
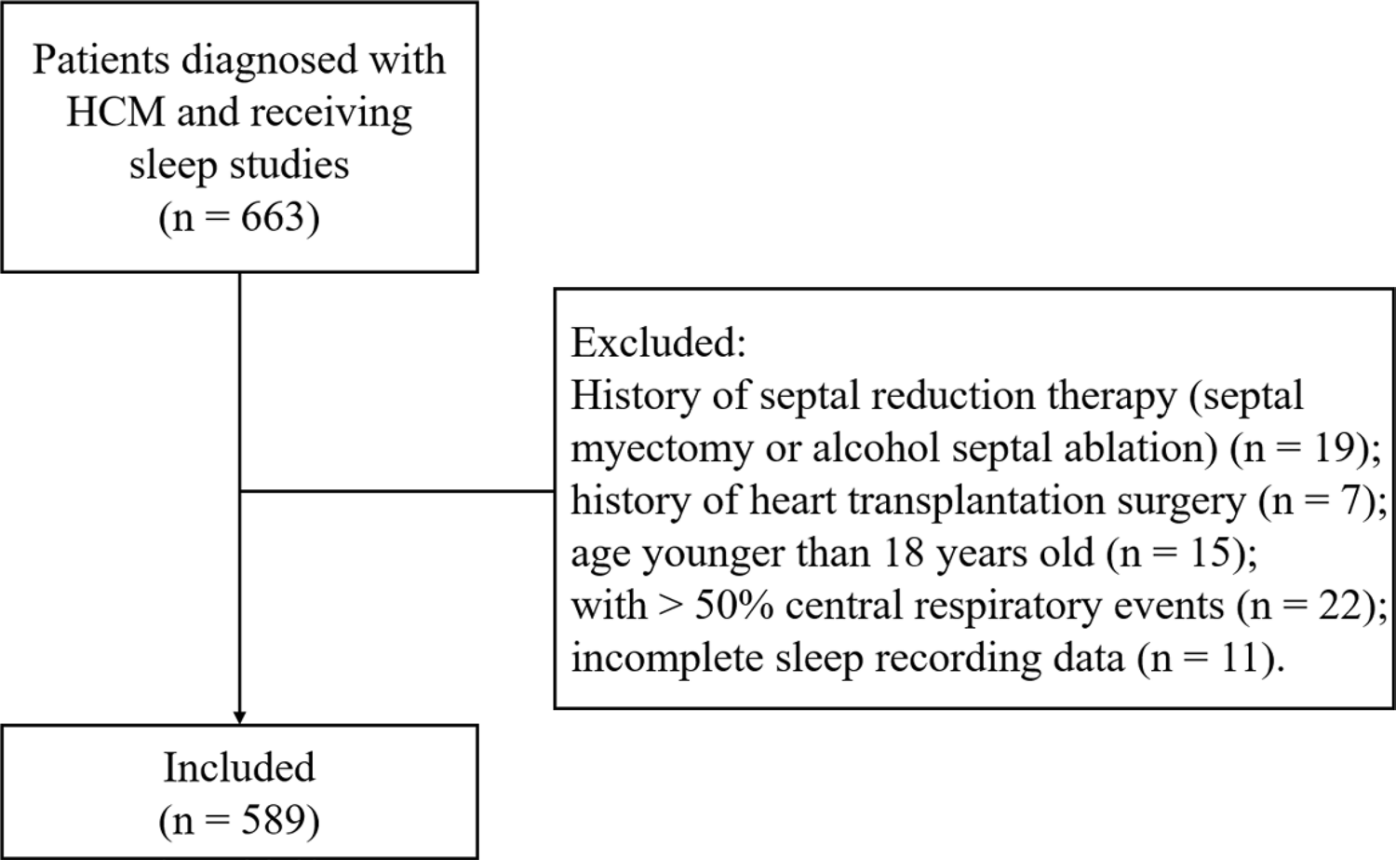
The study flowchart.

This study population were included as previously described^5^. The study flowchart was shown in Fig. 2. Briefly, between February 2010 to February 2019, patients who were diagnosed with HCM and underwent the first overnight sleep examinations from in-patient department at Fuwai Hospital were retrospectively included. Diagnosis of HCM was made based on an unexplained maximal left ventricle wall thickness ≥ 15 mm in the absence of other cardiac or systemic diseases capable of producing that magnitude of hypertrophy. Diagnostic criteria of HCM were consistent with the 2011 American Heart Association/American College of Cardiology and 2014 European Society of Cardiology guidelines^1,22^. Patients who had sleep examinations were clinically stable and did not undergo changes in New York Heart Association (NYHA) class over the last 30 days. No patient underwent continuous positive airway pressure treatment before. Patients were also excluded if they had central sleep apnea, had incomplete sleep data, were younger than 18 years old, had underwent septal reduction therapy before (septal myectomy or alcohol septal ablation), or had history of heart transplantation. According to the exclusion criteria, a total of 589 patients were finally enrolled. Patient demographics and clinical data within the first 3 days of hospitalization were collected.

### Sleep study

Sleep examinations were conducted as previously described^5^. An overnight polysomnography was performed in all study populations using the portable monitoring system Embletta (Medcare Flaga, Reykjavik, Iceland). This device continuously recorded finger pulse oximetry, nasal airflow by an airflow pressure transducer, thoracic and abdominal movement, body position, snoring, heart rate and ECG, and has been validated against full polysomnography^23^. The sleep study started automatically 30 min after the subjects went to bed. Polysomnography data were manually scored using standard criteria^24^. Apnea was defined as a decrease of nasal airflow by more than 90%, lasting at least 10 s. If the airflow absence is accompanied by a persistent or enhanced inspiratory effort, the apnea event is considered as obstructive. Hypopnea was defined as a 50% or discernible decrement in airflow lasting 10 s with oxygen desaturation of 4%^25^. The apnea–hypopnea index (AHI) was calculated by dividing the total number of apneic and hypopneic episodes by the total sleep time. The oxygen desaturation index was defined as the number of oxygen level drops 3% from baseline per hour. Mean and minimal oxygen saturation (SaO_2_), average pulse frequency and snoring proportion were also recorded. Diagnosis of OSA was made when the AHI in the recorded study was 5 events/h or more, irrespectively to daytime OSA symptoms, which allowed objective evaluation of the disease severity^26^. The severity of OSA was measured based on AHI: none OSA (AHI < 5 events/h), mild OSA (5 < AHI ≤ 15 events/h) and moderate to severe OSA (AHI ≥ 15 events/h).

### Echocardiographic study

Transthoracic echocardiography was performed using Vivid 7 ultrasound systems (GE Healthcare, Horten, Norway) with a multifrequency phased-array transducer. Echocardiographic examinations were performed by experienced physicians. Recordings were stored digitally and analyzed offline by independent observers. Standard measurements were made on the average of 3 cardiac cycles according to established criteria of the American Society of Echocardiography^27^. The measurements of left ventricular (LV) volume, LV ejection fraction and left atrial diameter (LAD) were determined from the apical view using biplane Simpson's rule method. The thickness of the interventricular septum (IVST) and ventricular wall was determined during diastole. The representative thickness of the interventricular septum, which was usually the thickness of the point 25 mm under the right coronary sinus nadir, was recorded to indicate overall thickness. Left ventricular outflow tract (LVOT) gradient was measured in the apical views by continuous-wave Doppler echocardiography under resting conditions and during provocative maneuvers, such as Valsalva, treadmill exercise, and/or amyl nitrite inhalation, to elicit latent obstruction, as previously reported^28^. We defined obstructive HCM patients as who satisfied one of the following criteria: (1) rest LVOT peak gradient ≥ 30 mmHg or (2) rest LVOT peak gradient < 30 mmHg with provoked LVOT peak gradient ≥ 30 mm Hg. Patients with both rest and provoked LVOT peak gradient < 30 mmHg were defined as nonobstructive HCM.

### Statistical analysis

The results were expressed as mean ± standard deviation, median (interquartile range), or number (percentage). Continuous variables were tested for normal distribution with the Kolmogorov–Smirnov test. Differences of continuous variables between groups were compared using Student unpaired t test or Mann–Whitney U test, as appropriate. Comparison of categorical variables was performed using the χ^2^ or Fisher exact test, as appropriate. Univariate logistic regression analyses were used to determine the association between the clinical or demographic characteristics and the presence of OSA. The test for collinearity was done before fitting multivariate logistic regression to produce efficient multivariate models. Variables were included into multivariate analysis because of statistical significance in univariate analysis including male sex, age, BMI, cigarette use, hypertension, diabetes, coronary heart disease, stroke, atrial fibrillation, creatine, LVOT obstruction, LAD, LV end-diastolic diameter, IVST, the use of calcium channel blockers and angiotensin-converting enzyme inhibitors or angiotensin II receptor blockers. The multivariate model was adjusted for the above covariates in a forward stepwise way. Results are expressed as odds ratio (OR) and 95% confidence interval (CI). Receiver operating characteristics (ROC) curve and area under the ROC curve (AUC) were used to assess the ability of the selected clinical characteristics to identify the presence of OSA. All reported probability values were 2-tailed, and a *P* value of < 0.05 was considered statistically significant. SPSS version 24.0 (IBM Corp., Armonk, NY) was used for calculations.

### Ethics approval and consent to participate

The study was approved by the ethics committee of Fuwai Hospital and was conducted in accordance with the ethical principles stated in the Declaration of Helsinki. The written informed consent was provided by each participant.

## Data Availability

The data used and analyzed during the current study are available from the corresponding author on reasonable request.
